# Strengthening the Connection between Science, Society and Environment to Develop Future French and European Bioeconomies: Cutting-Edge Research of VAALBIO Team at UCCS

**DOI:** 10.3390/molecules27123889

**Published:** 2022-06-17

**Authors:** Marcia Araque-Marin, Fabio Bellot Noronha, Mickäel Capron, Franck Dumeignil, Michèle Friend, Egon Heuson, Ivaldo Itabaiana, Louise Jalowiecki-Duhamel, Benjamin Katryniok, Axel Löfberg, Sébastien Paul, Robert Wojcieszak

**Affiliations:** 1 Univ. Lille, CNRS, Centrale Lille, Univ. Artois, UMR 8181-UCCS-Unité de Catalyse et Chimie du Solide, F-59000 Lille, France; marcia-carolina.araque-marin@centralelille.fr (M.A.-M.); fabio.bellot@int.gov.br (F.B.N.); mickael.capron@univ-lille.fr (M.C.); franck.dumeignil@univ-lille.fr (F.D.); michele@gwu.edu (M.F.); egon.heuson@centralelille.fr (E.H.); ivaldo@eq.ufrj.br (I.I.J.); louise.duhamel@univ-lille.fr (L.J.-D.); axel.lofberg@univ-lille.fr (A.L.); robert.wojcieszak@cnrs.fr (R.W.); 2 Catalysis, Biocatalysis and Chemical Processes Division, National Institute of Technology, Rio de Janeiro 20081-312, Brazil; 3 Department of Philosophy, George Washington University, Washington, DC 20052, USA; 4 Department of Biochemical Engineering, School of Chemistry, Federal University of Rio de Janeiro, Rio de Janeiro 21941-910, Brazil

**Keywords:** bioeconomy, catalysis, glycerol, furanics, hybrid catalysis, lignin, polymers

## Abstract

The development of the future French and European bioeconomies will involve developing new green chemical processes in which catalytic transformations are key. The VAALBIO team (valorization of alkanes and biomass) of the UCCS laboratory (Unité de Catalyse et Chimie du Solide) are working on various catalytic processes, either developing new catalysts and/or designing the whole catalytic processes. Our research is focused on both the fundamental and applied aspects of the processes. Through this review paper, we demonstrate the main topics developed by our team focusing mostly on oxygen- and hydrogen-related processes as well as on green hydrogen production and hybrid catalysis. The social impacts of the bioeconomy are also discussed applying the concept of the institutional compass.

## 1. Introduction

Since its creation in 2006, the VAALBIO group, standing for valorization of alkanes and biomass, has constantly been conducting dynamic research at UCCS (Unité de Catalyse et Chimie du Solide–UCCS UMR CNRS 8181), located in Lille, on catalysis for biomass valorization. This topic is indeed a pillar of bioeconomy development not only in France but also in Europe. The VAALBIO team today comprises more than 40 researchers. The group is internationally recognized in the field of heterogeneous catalysis and catalytic processes [1]. Recently, the VAALBIO team extended its research areas to chemo-enzymatic catalysis [2], hybrid catalysis and, even more recently, to human sciences [3] to be able to measure the impact of the implementation of new sustainable processes in future biorefineries. In this Special Issue of *Molecules* dedicated to “Sustainable Chemistry in France”, a few examples of our recent results are presented. For the sake of clarity, this review paper is divided based on the catalytic process. In the first part, the valorization of biosourced platform molecules such as alcohols, polyols or furanics using different atmospheres (oxidative, reductive, or neutral) is presented. In the second part, biomass fractionation and green hydrogen production are discussed. The combination of enzymatic catalysis and chemo-catalysis to carry out hybrid catalysis may lead to a real breakthrough in the field of catalysis in the coming years. The most recent advances of our group in this field are detailed hereafter. Finally, the new concept of institutional compass, a tool to assess the impact of the new renewable processes is also presented.

## 2. Recent Works and Results

### 2.1. Reactions Involving Oxygen

#### 2.1.1. Selective Oxidation of Glycerol

**Figure 1:**
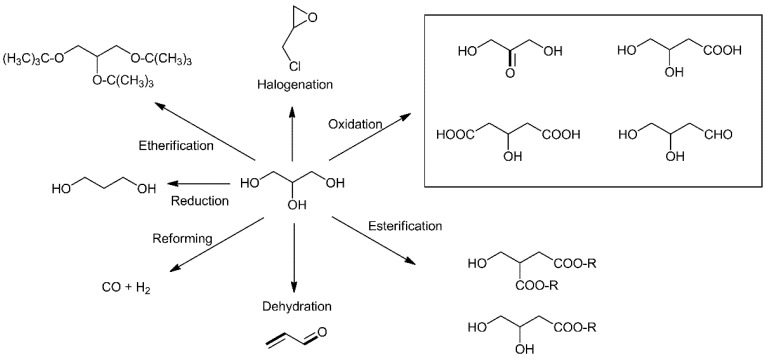
Examples of molecules of interest obtained through upgrading glycerol.

Glycerol is a waste product of biodiesel production. It is obtained as a byproduct of the reaction of the transesterification of triglycerides, which yields biodiesel (ca. 1 kg of glycerol is produced per 9 kg of biodiesel). Glycerol is considered as an important platform molecule due to (i) its high potential, thanks to the presence of three hydroxyl groups, which provide a high reactivity, and (ii) numerous upgrading possibilities (various target molecules) [4]. Figure 1 presents some of the molecules of interest that can be produced from glycerol such as epichlorohydrin through halogenation (epichlorohydrin), to diols as monomers for subsequent polymerization through reduction, acrolein which is a synthon that can be used to produce feed (DL-methionine) or super-adsorbent polymers for diapers (acrylates) through dehydration, and a variety of aldehydes, ketones or carboxylic acids of interest through selective oxidation to.

Among the aforementioned reactions, many studies have especially been published on the glycerol oxidation reaction in the liquid phase [5]. Most authors used noble metal-based catalysts (i.e., Pt, Au) in a basic medium to produce mainly glyceric and tartronic acids [6,7,8,9]. In our lab, mechanistic and kinetic studies have been carried out to compare the effects of various metals [10,11]. It has generally been observed that Au-based catalysts are only active in basic media, while Pt-based catalysts are more active and can be operated in a wide pH range (i.e., from basic to acid media). Because the price of glycerin strongly depends on its purity, we studied the effect of the different impurities present in crude glycerol [8]. The conclusion of such a study is that “impurities” such as methanol or the base used during the transesterification process help the oxidation of glycerol, but sulfides and MONG (i.e., Matter Organic Non-Glycerol: triglycerides and free fatty acids) hinder the catalytic performances drastically, mainly in terms of glycerol conversion, while selectivity is not impacted. Thus, prior to a catalytic reaction, it is highly recommended to at least remove MONG from crude glycerol, as it induces a strong deactivation of most catalysts by physically blocking their access to the active sites.

In the literature, only a few studies focusing on the production of glyceraldehyde from glycerol have been carried out [12,13,14], even though this aldehyde could be applied in many sectors (e.g., pharmaceuticals, cosmetics, etc.). Among the conventional metals used for glycerol oxidation in the liquid phase, only Pt shows a good activity toward glyceraldehyde formation. We performed our first study [15] on Pt/Al_2_O_3_ prepared using various methods, leading to various Pt nanoparticles sizes. We evidenced that in base-free conditions (but also in basic ones), selectivity is glycerol conversion dependent. Then, we dedicated a full study to the aforementioned reaction [16], keeping Pt as an active metal deposited on various supports and evidenced clearly that the selectivity toward glyceraldehyde is inversely linearly proportional to the glycerol conversion, irrespective of the support. Our lab-made Pt catalysts supported on SiO_2_, Al_2_O_3_ or TiO_2_ exhibited different initial glycerol conversion rates, highlighting them as good catalysts for the production of glyceraldehyde (i.e., Pt/TiO_2_). The Pt/TiO_2_ catalyst presents the smallest initial conversion rate and also the highest glyceraldehyde selectivity.

**Figure 2:**
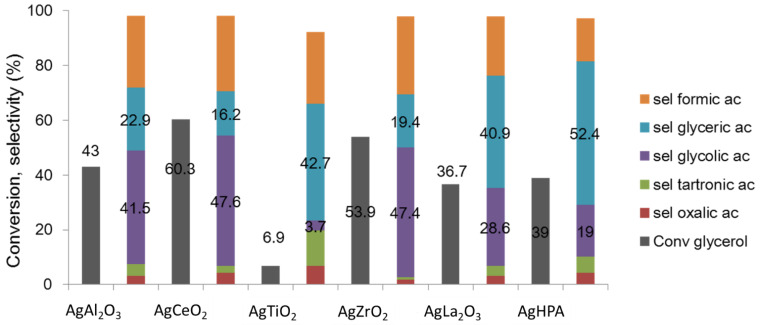
Glycerol oxidation using Ag-based catalysts. Reaction conditions: 5 h at 60 °C, 5 bar O_2_, NaOH/GLY = 4 (molar ratio), 0.3 M glycerol, 0.5 g catalyst, 1.4 wt.% Ag/support, and batch reactor.

Glycerol oxidation with C-C bond cleavage can lead to glycolic acid. This molecule finds applications as a tanning agent or in cosmetic formulations, for example. Only very few studies focus on the production of glycolic acid. This is due to the fact that over conventional noble metal-based catalysts under basic conditions, the formation of C3 acids is mainly observed (especially tartronic and glyceric acids). We demonstrated that by using Ag-based catalysts and choosing the ad hoc support, it is possible to favor the formation of glycolic acid [17,18]. Like for the aforementioned reaction, we first focused on a low glycerol concentration (i.e., 0.3 M) using a 1.4 wt.% Ag/Al_2_O_3_ catalyst. We chose different types of Al_2_O_3_ presenting different acid–basic characters (i.e., basic Al_2_O_3_, acidic Al_2_O_3_ and α-Al_2_O_3_). We showed that it is very important that the support presents a basic character in order to efficiently transform glycerol to glycolic acid. The evolution of glycolic acid yield follows the following order: basic Al_2_O_3_ > acidic Al_2_O_3_ > α-Al_2_O_3_ [18]. In order to optimize the catalyst formulation, we thus worked on different kinds of basic supports, keeping the other potential variables constant (e.g., the quantity of Ag, reaction conditions, etc.) [19]. As illustrated in Figure 2, the catalysts constituted of 1.4 wt.% Ag deposited on hydrotalcite (HPA) or on La_2_O_3_, well known for their basic characters, orientated the selectivity toward glyceric acid. In this figure, we can see that using ZrO_2_ and CeO_2_ allows us to increase the glycolic yield compared to the aforementioned basic alumina (first catalyst presented in Figure 2). The next step of the study was to prepare a new support by mixing the two aforementioned ones and increasing the quantity of silver [20], which led to an optimal composition, 5 wt.%/Ce_0.75_Zr_0.25_O_2_. Owing to this formulation, we worked on the optimization of glycolic acid productivity by increasing the initial glycerol concentration up to 2 M. Using a Design of Experiments (DOE) methodology, we thus optimized the reaction conditions in order to maximize glycolic acid productivity. We obtained 0.88 M of glycolic acid, starting from a 2 M glycerol solution. Increasing the initial glycerol conversion led to the need for an increase in O_2_ in the batch reactor.

#### 2.1.2. Allyl Alcohol Oxidation to Acrylic Acid

**Figure 3:**
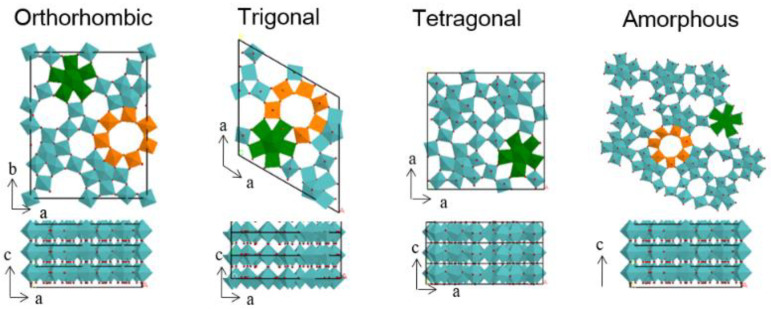
Structures of Mo_3_VO_x_ catalysts employed in the selective oxidation of allyl alcohol to acrylic acid.

Acrylic acid is one of the most commonly used intermediates used today, with USD 12 billion being spent on it in 2020. The selective oxidation of allyl alcohol to acrylic acid was studied over orthorhombic, trigonal, tetragonal and amorphous Mo_3_VO_x_ [21]. The orthorhombic and trigonal Mo_3_VO_x_ catalysts showed very similar catalytic performances, with an interesting shift in selectivity depending on the reaction temperature. In fact, at low temperatures (<200 °C), propanal and acrolein were found as the main products. Upon increasing the reaction temperature, the selectivity of the latter decreased gradually, giving rise to acrylic acid and propionic acid and yielding up to 66% acrylic acid at 350 °C for the orthorhombic Mo_3_VO_x_ catalyst and 68% at 325 °C for the trigonal Mo_3_VO_x_ catalyst. On the other hand, the tetragonal Mo_3_VO_x_ catalyst was much less active than the orthorhombic Mo_3_VO_x_ and trigonal Mo_3_VO_x_ catalysts, and exhibited a totally different product distribution, mainly yielding acrolein (>70%), even at an increased reaction temperature of 400 °C. Since all the studied catalysts have the same chemical composition, the difference in the observed catalytic activity was ascribed to the structure, meaning that the reaction is structure sensitive. In fact, Mo_3_VO_x_ possesses the same layer-type structure in the c-direction, but with a different arrangement of the pentagonal {Mo_6_O_21_} units in the a-b plane (Figure 3) [22]. Orthorhombic, trigonal and amorphous Mo_3_VO_x_ exhibit heptagonal channels, whereas tetragonal Mo_3_VO_x_ does not. The exposed section surface and the side surface of the rod-shaped crystals provide different active sites for oxidation reactions. Since the arrangement in the c-direction was identical for the four catalysts, it was concluded that the side surface of the Mo_3_VO_x_ catalysts was responsible for the selective oxidation from allyl alcohol to acrolein, whereas the heptagonal arrangement in the a-b plane catalyzed the oxidation of allyl alcohol to acrylic acid. It is further worth pointing out that the catalysts exhibited stable performances throughout the whole catalytic test cycle (>72 h), evidencing the high stability of the structure and the absence of any sintering or phase rearrangement, also shown by the XRD and N_2_-physisorption of the spent catalysts.

#### 2.1.3. Selective Oxidation of Furanics

Chemically speaking, furfural and 5-hydroxymethylfurfural are heteroaromatic molecules (furan ring) that also contain an aldehyde group. The transformation of these molecules into valuable chemicals is made possible by the presence of these functional groups [23]. The aldehyde group can be oxidized to an acid or undergo direct oxidative esterification. Below, we discuss the most important aspects that bring together works from the recent years of research on the catalytic transformations of furfural (FF) and 5-hydroxymethylfurfural (HMF) through oxidation processes. Given that Au-based catalysts have shown a high potential for achieving the aerobic oxidation of many substrates in water, their use in furfural oxidation would be advantageous [23,24,25]. However, the use of gold catalysts for the production of furoic acid, maleic acid, or succinic acid under uncontrolled pH conditions (without the addition of a base) is not well documented yet. Some of the first documents on this topic were published by our group [26,27,28,29,30,31,32,33,34,35]. Over the last 10 years of research on furfural oxidation, we have studied several factors that influence conversion and selectivity in this process. All works were performed in base-free conditions. In addition, we have also developed an in situ method to monitor the liquid phase oxidation of furfural via Raman spectroscopy [34]. Our first study focused on gold nanoparticles supported on different hydrotalcites [27]. It is known that the basicity of a hydrotalcite carrier strongly depends on the Mg:Al ratio used during synthesis. Hydrotalcite supports were prepared by a co-precipitation method using different Mg:Al ratios (5:1, 4:1, 3:1 and 2:1). All catalysts were active in the liquid phase oxidation of furfural at 110 °C and under 6 bar of oxygen. As expected, strong increases both in the furfural conversion and the yield to furoic acid were observed using supported gold nanoparticles catalysts as compared to the supports alone [27]. All catalysts were active in the oxidation of furfural in the liquid phase at 110 °C and under 6 bar of oxygen (Table 1). As expected, strong increases in both furfural conversion and furoic acid yield were observed using gold nanoparticle catalysts compared to the supports alone [27].

Complete conversion and 100% selectivity to furoic acid were achieved with Au/HT 4:1 and Au/HT 5:1 catalysts. This was probably due to the increase in the overall alkalinity of the support, which promoted the activity of the catalysts. No leaching of gold was detected; however, the Mg concentration in the post-reaction mixture steadily increased with reaction time, indicating gradual leaching. The pH of all samples after the reaction was 7, which was not expected since both furfural and furoic acid solutions have a pH of about 3 in water. This is strong evidence that a soluble base was formed in situ [27]. Our works showed that the presence of MgO is essential to achieve a high activity of the catalysts due to the in situ -OH formation. However, the dissolution of the support is an important issue. We tried to find a way to stabilize the MgO on the catalyst and to prevent the leaching. Thus, we proposed a novel catalyst in which Au is supported on a Mg-based support, a MgF_2_-MgO mixed phase solid [29]. Au/MgF_2_ displayed a rather low catalytic activity, similar to those of titania-, zirconia- and ceria-based catalysts, although it was less prone to the formation of FA.

**Figure 4:**
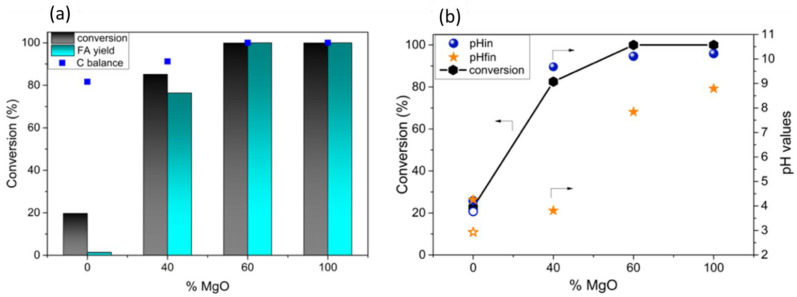
Base-free furfural oxidation using Au-based catalysts supported on the MgF_2_-MgO with various relative amounts of MgF_2_ and MgO. (**a**) Furfural conversion and FA yield versus % MgO; (**b**) furfural conversion and initial and final pH values versus % MgO (solid symbols) and blank test (open symbol) (49.4 μmol, substrate/metal = 50 (mol/mol), air (26 bar), 110 °C, 600 rpm, 2 h). pH_in_ indicates the pH measured before reaction (furfural solution + catalyst) and pH_fin_ indicates the pH measured after reaction. Reprinted with permission from Ref. [29].

The presence of the catalyst did not yield any change in the reaction pH, so there was no evidence of support leaching. Therefore, the degradation and/or side reactions and lower catalytic performance can be attributed to the acidic reaction medium, which does not favor Au-based solid catalytic functions. Decreasing the content of MgF_2_ in the mixed-phase (MgF_2_-MgO) support resulted in higher conversions, FA yields and carbon mass balances, as highlighted in Figure 4. Au/MgF_2_-MgO catalysts were very effective in the oxidation of furfural to FA, reaching a 99% yield after 2 h of reaction. A value of 60 mol % of magnesium oxide in the mixed system (MgF_2_-MgO) is enough to provide the same activity as that of pure MgO under the same conditions [29].

#### 2.1.4. Adipic Acid Synthesis

Adipic acid is one of the most important chemical intermediates—with a foreseen market of USD 8 billion in 2024—used in the preparation of nylon-6,6 polyamide, fibers and plasticizers. It is generally obtained using HNO_3_ in a two-step process. The oxidation process of cyclohexanone and cyclohexanol (KA oils) is seriously harmful to the environment because of the nitrous oxide (N_2_O) produced in huge amounts. It is generally thought to be a major factor of global warming and specifically of ozone depletion [36]. With this objective, we developed a new process which could be industrially applicable. The VAALBIO group is working on two different routes to obtain adipic acid. Cyclohexane was chosen as a starting reagent because it is largely available and not expensive.

**Figure 5:**
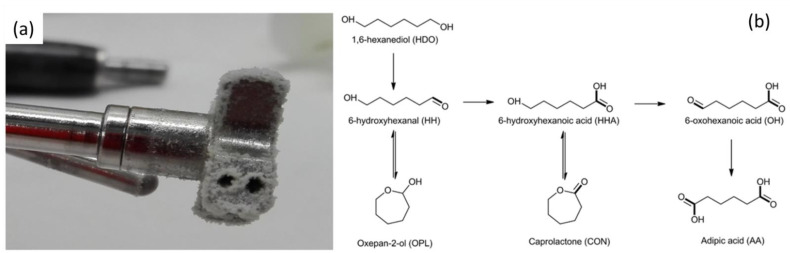
(**a**) Adipic acid formed from cyclohexane oxidation under solvent-free conditions; (**b**) adipic acid synthesis pathways from 1,6-hexanediol.

Moreover, unlike to cyclohexene, it can be oxidized using air under solvent-free conditions. Produced adipic acid is not soluble in cyclohexane and can be easily removed from the reaction media (Figure 5a). The highest AA selectivity and yield in a 1 mol scale reaction was obtained at 135 °C after 10 h of reaction using the VPO/CeO_2_ catalyst (Table 2).

**Figure 6:**
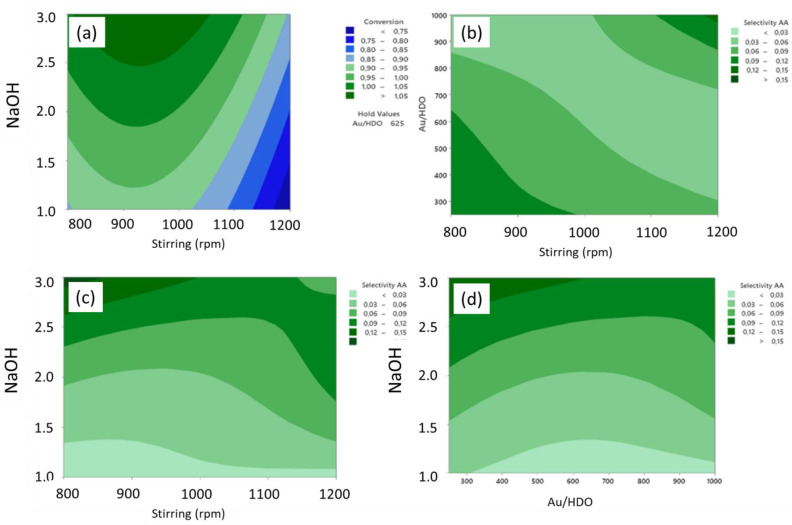
Contour plots of HDO conversion (**a**) and adipic acid selectivity versus (**b**) Au/HDO ratio and agitation, (**c**) NaOH equivalent and agitation, (**d**) NaOH equivalent and Au/HDO ratio.

Adipic acid can also be obtained through the oxidation of 1,6-hexanediol, as presented in Figure 5b. We have investigated this reaction using supported gold nanoparticles with different particle sizes and particle size distributions [37]. The mean diameters of the gold nanoparticles varied from 3 to 8 nm. Various experimental parameters were studied for the optimization of reaction conditions, such as the Au/HDO ratio, NaOH equivalent, agitation and temperature (Figure 6), by applying the DoE approach. We showed that the most important parameters are Au particle size, HDO to Au molar ratio and agitation. The best results in terms of the adipic acid yield were obtained using Au obtained via the sol-immobilization method with polyvinyl alcohol (PVA) catalysts (40% at 110 °C), however, with a low HDO to Au molar ratio (100). Under basic conditions and with a high HDO to Au ratio, the adipic acid yield was about 20%, with a much higher carbon balance (about 95%) [37].

#### 2.1.5. Selective Oxidation of H_2_S via Chemical Looping

The selective oxidation of H_2_S to elemental S is a very attractive alternative to the Claus process but suffers several drawbacks. The selectivity can be affected by catalysts’ efficiency and operating conditions, and catalysts usually suffer from deactivation. Another aspect to consider is that usually reactions must be performed at a low H_2_S concentration (typically a few % at most). Indeed, due to safety issues, a high concentration of H_2_S (mixed to oxygen) cannot be processed. Furthermore, the presence of oxygen does not allow the treatment of H_2_S in the presence of other combustible compounds, meaning that natural gas or biogas containing H_2_S cannot be treated directly. To overcome these drawbacks, in 2016, UCCS proposed an innovative approach for the selective oxidation of H_2_S to S using the “chemical looping” (CL) concept. Such processes involve an oxygen carrier which is reduced in the presence of a reductant and produces the desired product. Subsequently, the exposure of the reduced solid to an oxidant allows the re-oxidation and regeneration of the carrier. This is intrinsically a transient process which involves the use of circulating fluidized bed systems [38] or fixed-bed switching reactors. At the laboratory scale, the fixed-bed reactor, which is fed alternatively with the gas reactants, is usually the most appropriate, while circulating bed reactor systems are better adapted for industrial applications, and are already used in fluid catalytic cracking [39]. CL processes were initially proposed for total combustion but have since been studied for several reactions of industrial interest, such as the reforming reaction [40,41]. When applied for the selective oxidation of H_2_S to elemental sulfur, the concept of CL consists of separating the reaction into two distinct steps (1 and 2):

H_2_S + OCox → 1/*n* Sn + H_2_O + OCred (1)

OCred + ½ O_2_ → OCox (2)

where OCox and OCred represent the oxygen carrier in the oxidized and reduced forms, respectively.

The advantages of CL with respect to direct, co-feed, and oxidation reactions are the following: selectivity can be improved by avoiding a direct interaction between adsorbed sulfur species and di-oxygen from the gas phase or adsorbed onto the surface of the material and favoring the reactivity of lattice oxygen species; sulfur species deposited on the surface of the carrier can be removed during the regeneration step, thus avoiding the deactivation of the process; no mixing of H_2_S and O_2_ can improve the safety of the process and enlarge the range of H_2_S concentrations which may be processed; the preferential oxidation of H_2_S could be performed in the presence of other combustible gases such as methane.

In the last case, if the preferential oxidation of H_2_S is achieved in the presence of methane, then the direct purification of natural or biogas could be performed without the need of prior separation of H_2_S, presenting a major breakthrough in natural and biogas treatments.

The idea of applying chemical looping to the selective oxidation of H_2_S was proposed in 2016 through a patent application [42]. Since then, fundamental research has been performed on the topic and the validity of this concept has also been tested in industrial process conditions and in the presence of impurities. The key to such a development is in the oxygen carrier, which needs to fulfill specific requirements. It should have:

- A high reactivity in both the oxidation and the regeneration reactions;
- A good selectivity for the desired oxidation product, i.e., elemental sulfur;
- A good stability over many redox cycles;
- A good resistance towards sulfur deposition and removal.

Other properties such as a good mechanical resistance in circulating bed systems will be required for the industrial implementation of the process. V_2_O_5_ is well known for its oxygen mobility and storage capacity and catalytic reactivity for H_2_S oxidation [43]. It was therefore chosen as a potential oxygen carrier in the chemical looping mode after checking the thermodynamic feasibility of the process on this material. V_2_O_5_ proved to be highly reactive and selective towards elemental sulfur at rather low temperatures (150–200 °C) [44]. It could be seen that even at such low temperatures, sub-surface V species are involved in the looping process and not only surface species. On the contrary, it is suspected that uppermost surface species would be responsible for unselective SO_2_ production in the reductant step (i.e., during exposure to H_2_S). Depending on the reaction conditions, some deactivation of the system could be observed, but characterizations performed on used samples suggest that sulfur accumulation should not be responsible for this. The typical oxide materials used for supporting catalysts, such as TiO_2_, SiO_2_, CeO_2_, Al_2_O_3_ or ZrO_2_, have been considered for supporting the V_2_O_5_ active phase [45]. Some of these materials show their own redox capacity, which may interfere with the looping process, as in the case of CeO_2_. Otherwise, TiO_2_ and SiO_2_ have been identified as the most promising supports as they provide analogous or even better performances in comparison with bulk V_2_O_5_.

A deeper investigation into V_2_O_5_ supported on TiO_2_ [46] has shown that optimal performances could be reached for the carrier containing the equivalent of two monolayers of V_2_O_5_ dispersed on the surface of the support. Lower loadings tend to generate very reactive but less selective active species. Furthermore, as the absolute oxygen capacity of the carrier is dependent on the amount of V_2_O_5_, low-loading carriers rapidly reach the limit of H_2_S transformation. This induces adsorbed H_2_S to be converted to SO_2_ in the regeneration step. Furthermore, it may also lead to the over-reduction of the vanadium species, which are suspected to be less selective towards elemental S. For higher loading, the formation of tower-like V_2_O_5_ structures on the surface of the support seems to be detrimental to the reactivity of the carrier. Several phenomena may occur. First, larger crystallites exhibit a higher proportion of V^5+^ species (with respect to V^4+^), which are less reactive or less selective, in particular for surface species with respect to bulk ones. Second, in the case of tower-like structures, bare TiO_2_ can still be exposed, even at high loadings. This can lead to H_2_S adsorption generating higher amounts of SO_2_ produced during the oxidant (regeneration) step. Finally, the textural properties of the support have been proved to be very important for this process with respect to the selectivity towards elemental sulfur. A support with higher porosity may induce consecutive reactions in the reductant step and thus the over-oxidation of product, but also the accumulation of adsorbed sulfur species (e.g., elemental S produced, or unreacted H_2_S). These would then be oxidized in SO_2_ during the regeneration steps, contributing to low selectivity. The study performed on the optimal loading of V_2_O_5_ (two monolayers) onto TiO_2_ with a low surface area further confirms the importance of finding a good balance between vanadium species in different valence states. All results suggest that the best selectivity in elemental sulfur is obtained when V^5+^ to V^4+^ reduction occurs, whereas V^4+^ to V^3+^ reduction seems to be more favorable for unselective oxidation. On the other hand, highly oxidized systems with high proportions of V^5+^ seem to be less reactive and slightly less selective, as already mentioned. The optimal situation is when approx. 30% of V is converted from V^5+^ to V^4+^, but starting from materials which already contain significant proportions of V^4+^ (>30%).

### 2.2. Reactions Involving Hydrogen

#### 2.2.1. Carbohydrate Hydrogenation

**Figure 7:**
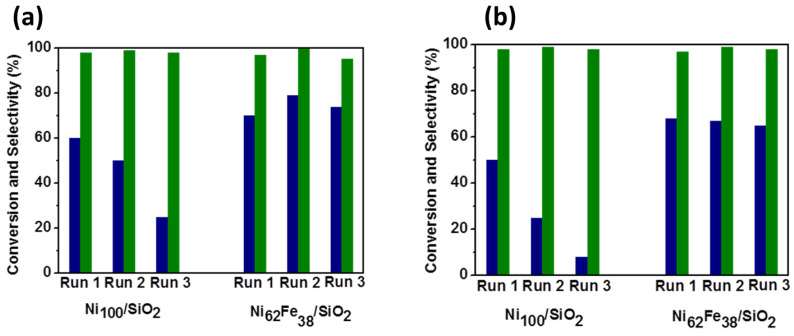
Catalyst recyclability study: (**a**) blue bars represent xylose conversion; green bars represent xylitol selectivity. Reaction conditions: Xylose—0.26 mol L^−1^; 20 bar H_2_; 80 °C; 108 mg catalyst; 90 min reaction time; n_xylose_/n_Ni_ = 9.7; n_xylose_/n_Ni_ + n_Fe_ = 10.6. (**b**) Blue bars represent maltose conversion; green bars represent maltitol selectivity. Reaction conditions: 8.8 wt.% maltose; 20 bar H_2_; 80 °C; 108 mg catalyst; reaction time = 8 h; n_maltose_/n_Ni_ = 9.8; n_maltose_/n_Ni_ + n_Fe_ = 10.0. Adapted with permission from Refs. [49,50].

The hydrogenation of the carbonyl groups of carbohydrates leads to the formation of polyols. Industrially, the three most important polyols are: sorbitol (from glucose) [47,48], xylitol (from xylose) [49] and maltitol (from maltose) [50]. In the case of xylitol, for example, the global industrial production is estimated to be about around 200 kt/year. The hydrogenation of xylose is usually performed in aqueous solutions using batch reactors at temperatures between 100 and 150 °C under high pressures of H_2_ (up to 50 bar). Comparable conditions are also applied in the case of glucose and maltose hydrogenations. Generally, these hydrogen-related processes are carried out on noble metals such as Ru, Pd, Pt and Ru, but Raney Ni is also used, especially in industrial applications. The VAALBIO group is working on different aspects of these processes: developing new catalysts, improving the stability of the existing catalysts and studying the kinetic aspects of the reaction. Recently, we studied new Ni-Fe-based catalysts for maltose and xylose hydrogenation in water. The Ni-Fe alloy is a good option to replace nickel catalysts. A Ni_62_Fe_38_/SiO_2_ bimetallic catalyst prepared by deposition–precipitation with urea (DPU) showed high activity in the hydrogenation of xylose and maltose in water [49,50]. We showed that the temperatures of 50–80 °C are most favored due to the limitation of the metal leaching and the presence of a kinetic regime even for high masses of the catalyst. The Ni_62_-Fe_38_/SiO_2_ catalyst exhibits a higher catalytic activity than the monometallic Ni catalyst. The selectivity to xylitol and maltitol (in the case of maltose hydrogenation) remained optimal and constant (~100%), even after three consecutive runs. The stability of the bimetallic catalysts was also better as compared to the Ni_100_/SiO_2_ catalyst (Figure 7), which showed a decrease in the catalytic activity after the first catalytic test, which is associated with a more pronounced formation of the initial Ni^2+^-phyllosilicate phase [49].

**Figure 8:**
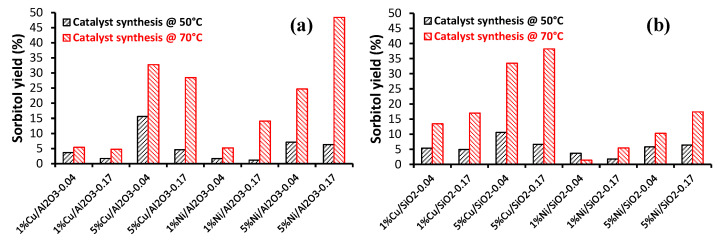
Sorbitol yields with (**a**) alumina-supported and (**b**) silica-supported catalysts synthesized at temperatures of 50 and 70 °C. (Test conditions: catalyst amount = 10 mg; 3 mL of glucose solution (1 wt.%); T = 130 °C; P_H2_ = 30 bar; t = 4 h). Adapted with permission from Ref. [48].

In the case of the glucose hydrogenation, we applied the high-throughput (HT) methodology for each step of the process: synthesis, characterization and catalytic testing. In total, 36 Ni and Cu catalysts were prepared by chemical reduction with the hydrazine method [48]. We chose the amount of Ni or Cu deposited on the support as the discriminative parameter. The results showed that the deposition of nickel and cooper on the oxide strongly depended on the nature of the support (SiO_2_ or Al_2_O_3_), the hydrazine/H_2_O ratio and the temperature of the reduction. The optimal conditions were as follows: high temperature (70 °C) and low N_2_H_4_/H_2_O ratio (0.04 mol/mol), irrespective of the metal precursors. The best catalytic results were obtained for the 5%Ni deposited on Al_2_O_3_ and 5% Cu deposited on SiO_2_ (Figure 8), synthetized at 70 °C.

#### 2.2.2. Furanics Hydrogenation

Quite a large number of metals can effectively catalyze furfural and HMF hydrogenation: Pt, Pd, Ru, Au, Rh, Ni, Cu, Co, and Fe [23,51,52,53]. A variety of products can be obtained. The selectivity of the transformation becomes, in this case, a key parameter. In most cases, the excessive hydrogenation of the furan ring and hydrogenolysis, which may involve (a) the alcohol group, (b) the opening of the heterocycle, and (c) the C-C bond of the ring-aldehyde, should be avoided [23,51]. When the transformation requires consecutive steps, the order in the hydrogenation/hydrogenolysis sequence must be carefully controlled. Thus, the key to realizing selective transformations is the proper tuning of the reaction system, both in terms of catalytic and experimental conditions. Most of the catalysts investigated in recent decades for the reductive conversion of FF and HMF are noble metal-based catalysts [54,55,56]. Noble metals show high hydrogenation activity, but their resources are limited. Base metals, such as iron, nickel and copper, are more available and less expensive. These systems can be optimized by combining them with other metals and by selecting the appropriate carrier, solvents, and/or co-catalysts (e.g., mineral acids).

Iron (Fe) and nickel (Ni) are two non-precious metals that can be used for the hydrogenation of organic molecules. While monometallic Ni is known to promote undesirable side reactions of hydrogenation and ring opening, Fe-Ni bimetallic catalysts have shown interesting properties for the selective hydrogenation of alcohol and aldehyde groups [57]. Fe-Ni/SiO_2_ catalysts were tested in the liquid-phase furfural hydrogenation reaction at 150 °C under 20 bar H_2_ [58,59]. The conversion of furfural increased rapidly with an increasing Ni content. The highest yield of furfuryl alcohol was obtained with Fe-Ni catalysts containing 60–75% at. Ni. An increasing Ni content systematically increases the consumption of furfuryl alcohol (FFA) through three parallel secondary reactions: etherification with isopropanol solvent, possibly catalyzed by metal ions that remain unreduced after activation. The formation rates of isopropyl furfuryl ether (iPrOMF), methylfurfural (MF), and tetrahydroxyfurfuryl alcohol (THFFA) from FFA also increased with Ni content. For catalysts with Ni content in the active phase lower than 84 wt.%, FFA is mainly consumed in the hydrogenolysis reaction to MF, in the slower etherification reaction to iPrOMF, and in the furan ring hydrogenation reaction to THFFA. For catalysts with a Ni content in the active phase higher than 84 wt.%, FFA is consumed mainly by etherification to iPrOMF, by the slower second hydrogenolysis route to MF and by the even slower hydrogenation of the furan ring to THFFA [59].

**Figure 9:**
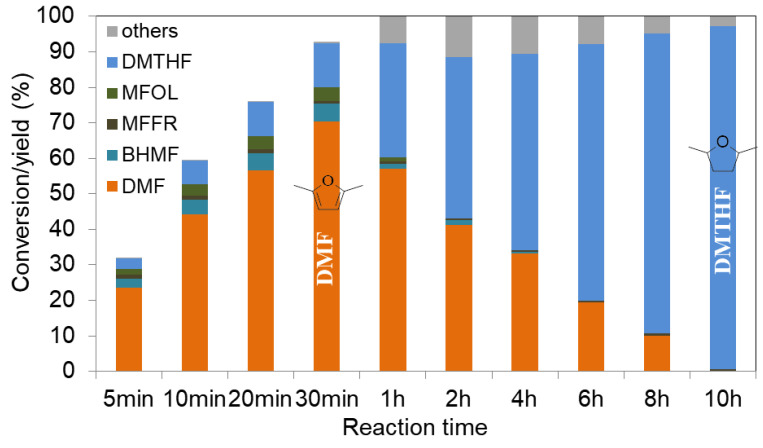
Evolution of HMF conversion and product yields with reaction time over Ni/SBA-15. Reaction conditions: 0.144 mmol/mL HMF in 25 mL 1,4-dioxane; P(H_2_) = 30 bar; T = 180 °C; HMF/Ni molar ratio of 3. Reprinted with permission from Ref. [60].

Nickel catalysts were also studied in HMF hydrogenation. We showed that a monometallic Ni/SBA-15 catalyst, prepared by incipient wetness impregnation, is able to convert HMF to 2,5-dimethylfuran (DMF) and 2,5-dimethyltetrahydrofuran (DMTHF) at 180 °C. This process occurs in two consecutive steps [60]. By controlling the reaction time, high yields to DMF (71%) or DMTHF (97%) at high conversion (97–100%) can be obtained (Figure 9).

Kinetic studies suggest that 5-hydroxymethylfurfural is converted via MFFR as an intermediate. It was shown that the C-O hydrogenolysis and C=O hydrogenation routes are much faster than the furan ring hydrogenation.

#### 2.2.3. Allyl Alcohol Synthesis from Glycerol

**Figure 10:**
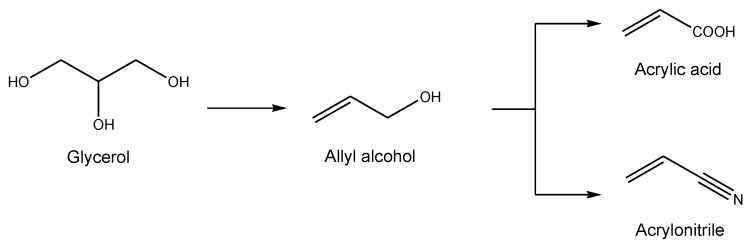
Production of important intermediates of the chemical industry using allyl alcohol from glycerol as an intermediate.

The use of sustainable resources for the synthesis of olefinic compounds is of much interest due to the importance of the latter as platform chemicals. As example, allyl alcohol is a promising platform molecule due to its broad range of applications. Its derivatives are present in resins, pharmaceutical products, perfumes and food formulations. Furthermore, allyl alcohol is considered as a promising starting material of important intermediates for the chemical industry, such as acrylonitrile and acrylic acid, which have large-scale applications for polymers and resins. Hereby, UCCS has developed catalysts for the whole value chain, starting from glycerol to acrylic acid and acrylonitrile via allyl alcohol as an intermediate (Figure 10).

**Figure 11:**
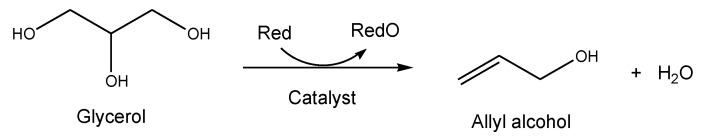
General reaction pathway for the deoxydehydration reaction. Red = reductant. RedO = oxidized reductant [66].

In fact, among the various bio-based pathways to allyl alcohol, those starting from glycerol are particularly interesting since it is the main by-product of the biodiesel industry. Glycerol is considered as a potential biorefinery feedstock to develop various processes of valuables chemicals such as glyceric acid, acrolein 1,2-propanediol and polyglycerol [61,62,63,64]. With the growing demand for sustainability, there has been increased attention on glycerol conversion toward less oxygenated derivatives, making it a preferred starting material for the synthesis of allyl alcohol. The transformation of glycerol to allyl alcohol has been focused on selective oxygen removal via the deoxydehydration reaction (DODH), a very competitive methodology which has been poorly investigated. The DODH reaction consists of the removal of two vicinal hydroxyl groups from a diol or polyol to yield the corresponding alkene (C=C bond). Figure 11 displays the case of the DODH of glycerol to allyl alcohol [65]. This reaction requires a stoichiometric reductant (red), which is oxidized during the reaction (redO).

An array of catalytic processes of the DODH of glycerol have been reported in the literature, including homogeneous [67] and heterogeneous catalysis [66,68,69]. The identification of an optimal reductant/catalyst couple has been a focus of research at UCCS. A catalyst for the DODH of glycerol to allyl alcohol using 2-hexanol as hydrogen was developed based on alumina-supported rhenium oxide [66]. The main drawback of this catalytic system is the co-produced ketone, namely, 2-hexanone. This molecule has a high toxicity and no profitable value can be envisaged. Consequently, the alcohol has to be regenerated in order to avoid ketone accumulation. Thus, a ceria-supported rhenium oxide catalyst was developed in combination with an alternative secondary alcohol-methyl isobutyl carbinol (MIBC). The employed mesoporous ceria support was synthetized via a nanocasting process using SiO_2_ and activated carbon as hard templates and impregnated with ReOx [70]. The resulting catalysts give excellent yields of up to 86%, with MIBC as a hydrogen donor and solvent, forming not only allyl alcohol as a valuable product, but also a stoichiometric amount of methyl isobutyl ketone (MIBK), a valuable organic solvent used in liquid–liquid extraction.

### 2.3. Reactions under Specific Atmosphere (CO_2_, NH_3_ and N_2_)

#### 2.3.1. Direct Carboxylation

**Figure 12:**
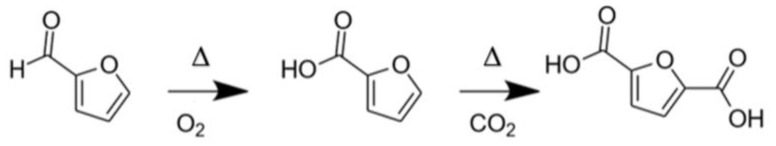
Two-step FDCA synthesis method of furfural using oxidation and carboxylation steps.

CO_2_ is also a valuable substrate for the synthesis of high-added-value molecules. The direct catalytic and sustainable carboxylation of aromatic molecules using a “simple” insertion of carbon dioxide into the sp2 C-H bond is highly desirable. In 1860, Kolbe reported the direct carboxylation of phenol with CO_2_ (Kolbe–Schmitt synthesis) [71]. Kolbe and Schmitt showed that the phenol group plays a crucial role in this reaction. Since then, various carboxylation processes have been studied. The most important are: (i) the Henkel reaction, (ii) enzymatic reactions, (iii) molten salts, (iv) homogeneous catalysis and (v) photo-electrocatalytic processes. However, many challenges are faced in these processes [35]. 2,5-Furandicarboxylic acid (FDCA) is a very important building block for the synthesis of polymers such as poly(ethylene furandicarboxylate) (PEF). PEF is generally proposed as an alternative to poly(ethylene terephthalate) (PET), a non-sustainable monomer [72]. The possibility of FDCA production from hemicellulose is of high interest from an industrial point of view. Furfural, which is already implemented in industrial production from non-edible resources, could act as a substitute for HMF. As illustrated in Figure 12, the catalytic oxidation of furfural forms 2-furoic acid. It can then undergo the direct C-H carboxylation with CO_2_ to produce FDCA [72,73]. These two catalytic processes can be performed sequentially (Figure 12) [72,73].

However, the main challenge of this process is inserting the carboxylate group into the C-H bonds [72]. CO_2_ is a C1 feedstock and presents kinetic and thermodynamic limitations [35,73]. Recently, we proposed a proof-of-concept process using heterogenous catalysts for the direct carboxylation of furoic acid using Henkel reaction conditions (devices, temperature, and catalyst/reactant ratio). The use of the Henkel reaction to selectively produce FDCA from furoic acid salt (K_2_F) using CdI_2_ as a catalyst has already been reported [72]. However, the minimum reaction temperature is 260 °C. At this, CdI_2_ starts to decompose, which improves the interaction between the semi-melted catalyst and the solid K_2_F. In order to reduce the working temperature, a heterogeneous catalyst was applied in addition to the CdI_2_ co-catalyst (Table 3).

Using a dual catalytic system, the reaction temperature was decreased to 230 °C since the K_2_F conversion did not change. The FDCA yield was not significantly affected when using both catalysts. When a 2 M HCl solution was applied to extract the crude product from the reaction medium, a higher STY yield was obtained. In addition, using the heterogeneous Ag/SiO_2_ catalyst permitted us to decrease the temperature to 200 °C [72].

#### 2.3.2. Ammoxidation

The ammoxidation of allyl alcohol to acrylonitrile is the subject of only very few studies, notably due to the importance of acrylonitrile as an intermediate (USD 11.8 billion in 2019). In 2013, our team reported the use of antimony-iron catalysts of different Fe/Sb ratios (0.6 and 1) for the ammoxidation of acrolein with 36% yield [74]. The application of the same catalyst was then studied in the ammoxidation of allyl alcohol, yielding up to 86% acrylonitrile under optimized reaction conditions (0.16 s contact time, 450 °C, AllylOH/O_2_/NH_3_ ratio 1/3.5/3) [75]. Surprisingly, both catalysts showed very similar performances despite the different Fe/Sb ratios. A detailed study of the surface composition by X-ray photoelectron spectroscopy (XPS) and low-energy ion scattering (LEIS) revealed that the surface layer of the catalyst was enriched in antimony after testing. The latter was ascribed to the formation of a FeSbO_4_ mixed phase under reaction—which was also evidenced by an induction period of the catalytic test with the increasing formation of acrylonitrile during the first hours on stream.

#### 2.3.3. Glycerol Polymerization

Glycerol polymers are highly demanded by industry. Different types of glycerol polymers can be obtained and thus directed to various target applications. Solid bases used as catalysts give the best performances, and, among them, the Ca-based catalysts are particularly considered as efficient [63]. Based on the analysis of spent calcium oxide and calcium hydroxide solids (X-ray powder diffraction and 13C solid-state NMR), we evidenced that the active phase is actually not the initial solid added to the medium, but rather a Ca-glycerolate phase (linear Ca(C_3_H_7_O_3_) or cyclic-branched Ca(C_3_H_6_O_3_)) formed in situ [76]. We explained the formation of Ca-glycerolate, e.g., from CaO through a dissolution and precipitation followed by crystallization.

#### 2.3.4. Butadiene Synthesis from Ethanol

1,3-Butadiene (1,3-BD) is a chemical intermediate of high interest in industry (USD 44.8 billion foreseen market value in 2026), especially due to its major application as a monomer for manufacturing synthetic rubber. However, the 1,3-BD supply and sustainability are at stake and alternative production routes are desired to at least partly get rid of the steam cracking of naphtha dependency. Realizing 1,3-BD production from bioethanol would fully unlock the value chain and enable a sustainable supply while, if correctly handled, yielding a lower environmental impact [77,78]. By combining screening methods to design experiment to optimize the catalytic formulations, [79] we rationalized the design of highly productive catalysts. Lab-made Zn-Ta-TUD-1, with Zn:Ta (mol:mol) between 1.5 and 2 and presenting a high specific value of >600 m^2^·g^−1^ with an average pore diameter of around 10 nm, was found to be very efficient [80]. With such a catalytic formulation, it was possible to realize the remarkable 1,3-BD productivity of 2.45 g 1,3-BD g_cat_ h^−1^ with performances over 60 h being much more stable than those of conventional catalysts. We found that the active phase is made up of highly dispersed Zn(II) species together with tetrahedral isolated species and monolayered clusters of Ta(V) (FTIR, UV-Vis-DRS, XPS, XRD and HR-STEM) [81].

### 2.4. H_2_ Production from Bioresources (Biomass and Biogas)

Hydrogen is an important chemical product, as the most used gas in the industry and also for the future “green” energy, that can be used directly by combustion or in combination with a fuel cell, leading to electricity. Today, H_2_ is mainly produced from fossil resources; it is urgent to produce it from renewable resources, not only so it can really be “green” but also to follow its increasing demand. Even if some H_2_ sources have been recently found, hydrogen is mainly found in numerous molecules (such as the simplest one, water, hydrocarbons, alcohols, etc.), so H_2_ needs to be produced. Therefore, at the international level, hydrogen production is largely studied, with the objective to use renewable resources and/or renewable energies. In this context, H_2_ production from ethanol (easily obtained from biomass) and from CH_4_ (biogas) are the subject of much research. To perform these reactions, noble metals (Pd, Pt, and Rh) and transition metals (Ni, Co, and Fe) with different bulk and supported catalysts have been extensively studied. Research has revealed that the redox properties and the strong metal–support interactions are the two key parameters to avoid sintering and carbon formation. Our objective was the development of ex-hydrotalcite (HT) compounds to enable us to incorporate metal cations for the selective production of hydrogen. Transition metal catalysts were preferred compared to the much more expensive noble metals ones, where Ni catalysts showed the best performances [82].

In the laboratory, H_2_ production is studied from ethanol by steam reforming (SRE), partial oxidation and oxidative steam reforming (OSRE, including autothermal reforming) reactions [82,83,84,85,86], and from CH_4_, by dry reforming (DRM) and oxidative dry reforming (ODRM) reactions [40,87,88,89,90]. These last reactions are of particular interest because they allow the transformation and valorization of two greenhouse gases (CH_4_ and CO_2_), and also can be applied to fuel cells [90]. Different types of Ni-based catalysts such as CeNi_x_Al_0.5_H_z_O_y_ (0 < x ≤ 5, z = 0 or 0.5) [40,82,83,87,88,89] and Ni_x_Mg_2_AlO_y_ (0 < x ≤ 12) [82,83,84] compounds were developed, with the objective to obtain highly performant, selective and stable catalysts. Ni-based catalysts are particularly interesting due to their ability to promote C-C-bond cleavage, participation in the water gas shift reaction and high performance in methane reforming. The influence of different parameters was analyzed to optimize the catalytic material (preparation, formulation, Ni content, and pretreatment) and the operational conditions (reactant concentration, reaction temperature, presence of O_2_ in the feed, etc.), including the chemical looping reaction [40,87,88,89].

**Figure 13:**
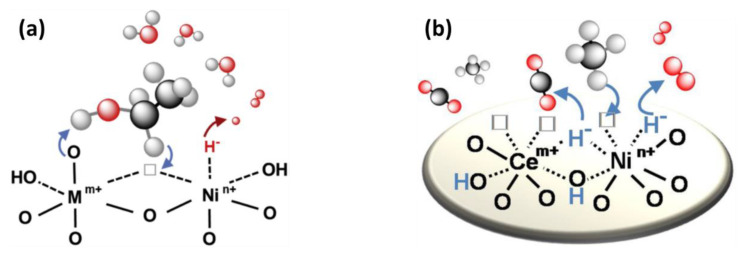
(**a**) OSRE on Mg_2_AlNi_x_H_z_O_y_ oxyhydride catalysts. Ni^n+^: Ni^2+^ or Ni^δ+^, M^m+^: Mg^2+^ or Al^3+^ and ☐: anionic vacancy. The positions of anionic vacancy and hydride species are arbitrary [85]; (**b**) ODRM on CeNi_x_Al_0.5_H_z_O_y_ oxyhydride catalysts. Ni^n+^: Ni^2+^, Ni^δ+^, Ce^m+^ = Ce^4+^, and Ce^3+^ (Al^3+^ can replace a cerium cation in the solid solution). ☐: anionic vacancy, red balls: oxygen, black balls: carbon, white balls: hydrogen. The positions of anionic vacancy and hydride species are arbitrary [89].

Optimizing the proportion of Ni cations (Ni^2+^) in strong interactions with other cations and the presence of anionic vacancies in the mixed oxides allow the formation of oxyhydride compounds, leading to particularly interesting material properties and mechanisms, involving the heterolytic abstraction of hydride species from ethanol (Figure 13a) [83] and from CH_4_ (Figure 13b) [89].

For example, using these properties, we developed particularly efficient cerium and Ni-based catalysts for ethanol transformation at room temperature, as well as a new technology, in the presence of oxygen in the feed [82]. Mg_2_AlNi_x_H_z_O_y_ nano-oxyhydrides were also shown to be highly performant in SRE and OSRE with high stability [86]. Carbon formation is decreased and a much lower input of energy of 50 °C is used to reach a temperature of 300 °C when O_2_ is added. Moreover, a higher production of H_2_ can be achieved at a reaction temperature of 300 °C in OSRE conditions compared to SRE (60 instead of 10 L h^−1^ g_cat_^−1^), mainly because of the beneficial use of a high concentration of ethanol (14 mol. %) in the presence of O_2_. For H_2_ production from methane, CeNi_x_Al_0.5_H_z_O_y_ (0.5 ≤ x ≤ 5) nano-oxyhydride catalysts were shown to be highly performant in ODRM (CH_4_/CO_2_/O_2_/N_2_ = 1:0.7:α:N_2_ with 20% of CH_4_ and α varying from 0 up to 0.5, 96,000 mL h^−1^ g_cat_^−1^) [89]. At 500 °C, the CO_2_ conversion overcomes the predicted value and the conversion of CH_4_ reaches the thermodynamic limit. At 600 °C, with an O_2_/CH_4_ ratio of 0.3, the CeNi_x_Al_0.5_H_z_O_y_ catalyst allows CH_4_ and CO_2_ conversions of about 63% and 50%, respectively, and a H_2_/CO ratio of about 1.3, without carbon formation.

### 2.5. Biomass Fractionation

Lignocellulosic biomass is an abundant, non-edible and renewable resource for the production of high value-added chemicals and fuels in future biorefineries. However, the use of this sustainable raw material as a feedstock requires the development of new chemical processes and catalysts. The conversion of lignocellulosic biomass into chemicals and fuels in a biorefinery involves a multi-step process: the fractionation of lignocellulosic biomass into its main components (cellulose, hemicellulose and lignin), followed by depolymerization and upgrading. These steps are performed separately or consecutively, which increases the costs. In addition, lignin is most often considered to be a waste and is burned for energy generation in first-generation biorefineries. These goals can be achieved with novel fractionation technologies that operates at low temperatures, which opens up the possibility for the integration of chemical and biological catalysis and the use of hybrid catalysts, in which chemical and biological catalysts work in tandem in one-pot and one-step reactions. However, these alternative processes require the design of more efficient and selective catalysts. Recently, a two-step processing of lignocellulosic biomass was previously carried out using acidic molten salt hydrate [91]. Inorganic molten salt hydrates are concentrated inorganic salt solutions with a water to salt molar ratio close to the coordination number of the strongest hydrated cation, with the water molecules bound to the inner coordination sphere of the cation, while the anion is free in solution [92,93,94]. They are inexpensive, easy to prepare and environmentally friendly as no toxic and volatile organic compounds are required to prepare such solvents. Moreover, the inorganic salts can be recovered by evaporating the water after cellulose regeneration and can be recycled for further use [95].

**Figure 14:**
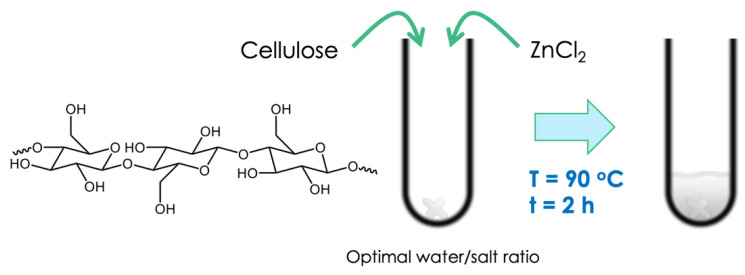
Example of cellulose hydrolysis using molten salt methodology.

Inorganic molten salt hydrates are known to dissolve cellulose, which depends on the water content as well as the structure of the cation coordination sphere [92,95,96,97]. The interaction between the cation and cellulose weakens and breaks the intra- and intermolecular hydrogen bonds of the cellulose chains. Different inorganic molten salt hydrates have the ability to dissolve cellulose (Figure 14), such as: LiBr, LiCl, ZnBr_2_, ZnCl_2_, FeBr_2_, and FeCl_3_ [92,97]. The dissolution of cellulose in inorganic molten salt hydrates decreases its crystallinity, which improves the reactivity of cellulose. Various inorganic molten salt hydrates have been used for cellulose hydrolysis under mild conditions (low acid concentration and temperature) [95,98,99]. The hydrolysis rate and product distribution are affected by the type and concentration of the inorganic molten salt hydrate, reaction temperature and the concentration of acid. Glucose, fructose, levoglucosan, HMF and levulinic acid are the major products reported, with the yield of each product depending on the type of molten salt hydrate. Once cellulose and hemicellulose are extracted from the biomass matrix, they are transformed into platform molecules by using a heterogeneous catalyst. Different commercial solid acid catalysts with various acidity values could be used (Amberlyst, Dowex, Nb_2_O_5_, NbPO_4_, ZrO_2_, SO_3_-ZrO_2_, ZSM-5, and USY). A catalyst containing highly active transition metal nanoparticles and acid sites deposited on an inorganic support was designed. The reaction involves the hydrolysis of the carbohydrates on the sulfonic groups grafted onto the silica support followed by the hydrogenation of the sugars produced on the metal (Ru and Pd) particles into sorbitol and xylitol, which are platform molecules. Lignin remains in the solid residue produced and it is separated and catalytically transformed into hydrocarbons by hydrodeoxygenation.

### 2.6. Hybrid Catalysis

In recent years, industrial biotechnology has had a significant impact on the pharmaceutical and chemical industries, backed up by advances in the field of enzyme evolution aimed at building new enzymes and catalysts. This has led to the development of more efficient manufacturing processes, while producing less waste and avoiding the use of harmful solvents and reagents [100]. Consumer demands for green and sustainable products with minimal environmental impact have been one of the biggest drivers of finding more sustainable routes in order to obtain compounds of great industrial value. Within this frame, chemical catalysis and enzymatic catalysis have moved closer to one to another, towards the innovative interdisciplinary concept of hybrid catalysis that aims to couple these different “worlds” in the same reactor [101]. This enables the improvement of reactions’ performances and yields new synthesis pathways through the combination of the best characteristics of each system, which must be first optimized separately before merging [102,103,104]. Such an approach is still rather scarcely developed at the international scale, while considerable growth can be expected in the mid-term. In this context, we are involved in some pioneering works. While such a combination has been long considered as inconceivable, we have demonstrated its relevance through a few different variations of the concept among the scope of possibilities it opens [2,105]. Hereafter is an overview of the achieved results in the field of hybrid catalysis by the VAALBIO team.

#### 2.6.1. Enhanced Dynamic Kinetic Resolution

**Figure 15:**
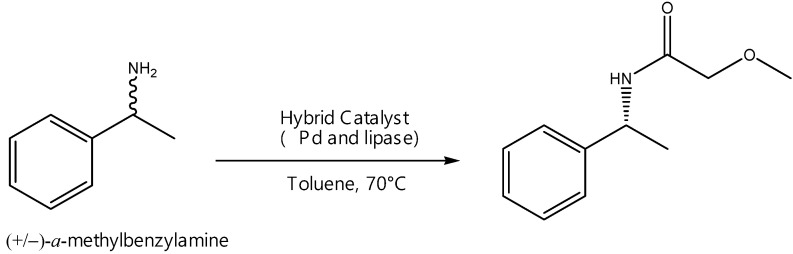
Example of DKR reaction using hybrid Pd-lipase system [111].

Among the various strategies for separating two enantiomers in a racemic mixture, the kinetic resolution (KR) and the dynamic kinetic resolution (DKR) have been of great importance in global chemistry [106]. In a KR, enantiomers react with different reaction rates and selectivities in reaction with a catalyst, biocatalyst or chiral reagent, resulting in an enantio-enriched sample of the less reactive enantiomer. However, a crucial limitation of KR is that the maximum theoretical yield is 50%. One solution is the combination of a chemical catalyst capable of racemizing the unwanted enantiomer in situ. In this way, the racemate obtained returns to the KR process [107,108]. This process is named dynamic kinetic resolution (DKR) and a theoretical yield of 100% can be achieved. This strategy has been successfully applied to a wide range of other compounds, such as chiral amines. Generally, racemization requires thermal or strongly acidic or basic conditions that are incompatible with a stereo-transformation into a single batch reactor or in continuous processes. On the other hand, several transition metal complexes such as rhodium, ruthenium and iridium are known to catalyze the racemization of optically active amines under mild conditions [109]. One solution to this is the use of hybrid catalysts, which involves cooperation between an enzyme and metal catalyst [110]. Among the numerous enzymes that have been applied in DKR for obtaining enantiomerically pure amines, lipases (triacylglycerol hydrolases, *E.C. 3.1.1.3*) are the most widespread group [110]. A number of published procedures that take advantage of a reagent/chemical catalyst chemical to perform dynamic kinetic resolution have been reported (Figure 15).

For example, supported Pd nanoparticles with immobilized lipases have been shown to facilitate this reaction, as shown below (Table 4) [111].

In this work, lipase CaLB was immobilized on functionalized Pd-SiO_2_ nanoparticles containing Pd in order to simplify the DKR of α-methylbenzylamine. As a result, ee >99% and conversion of 82% were found, with only 1% of Pd, generating a productivity of 2.21 mg of product h^−1^ mg of support^−1^ against 0.76 found for N435^®^. Compared to commercial N435^®^, the novel biocatalysts showed protein loads about 15-fold lower and higher activity, demonstrating competitive performances and good industrial applications [111].

The biggest challenge in hybrid catalysis is to combine the best conditions of each system. The hybrid catalyst was around 50−100 nm with nanoparticulated Pd (5−10 nm) on its surface, and presented a superparamagnetic behavior without magnetization and 22 emu g^−1^ of saturation magnetization [112]. As a result, it was possible to achieve a 99% conversion, with 95% selectivity and 93% enantiomeric excess after 12 h in batch. For a continuous flow system, it was possible to achieve a 95% conversion, with 71% selectivity and an ee > 99% after 60 min of reaction. Because the fixed-bed reactor system is pressurized, ammonium formate was used in this work as an in situ hydrogen source (Table 5) [112].

As a result, a magnetic hybrid biocatalyst was able to promote high conversions and selectivities, close to systems where commercial lipase N435 was used as the enzyme source. Thus, the newly constructed hybrid biocatalyst becomes an interesting and robust alternative for obtaining chiral blocks containing amines [112].

We additionally developed two hybrid catalysis-based approaches to optimize the conversion reaction of d-glucose to 5-hydroxymethylfurfural (HMF). HMF is a platform molecule [113] opening the way to the synthesis of many compounds with applications in the fields of biofuels, biopolymers and a whole range of fine chemicals, making it one of the most studied platform molecules. To obtain HMF from d-glucose, the latter must first be isomerized to d-fructose. However, this reaction suffers from an unfavorable thermodynamic equilibrium which results in the presence of both sugars in almost equal quantities. An effective strategy consists of carrying out the isomerization with the aid of an enzyme and a d-glucose isomerase, and coupling this to the chemical dehydration step, making it possible to achieve reaction equilibrium using a hybrid catalytic process. Several attempts have already been made in this respect, and we present below two of our developments using hybrid catalysis, one involving a classical enzymatic isomerization step coupled with an innovative compartmentalization approach to shift the equilibrium toward the products formation and an innovative hybrid isomerization step based on the combination of another enzyme and a chemo-catalyst for the regeneration of its co-substrate.

#### 2.6.2. Compartmentalization

**Figure 16:**
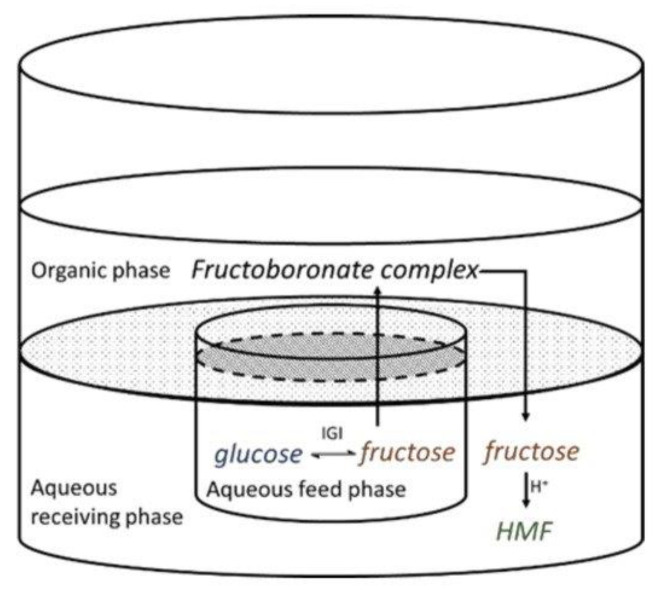
Concept of the compartmented sequential reactor with an organic solvent as the liquid membrane [116].

The compartmentalization based on the cascade reaction was the first approach studied in our laboratory [114,115,116]. This method is based on the subsequent dehydration of D-fructose to HMF using a Brønsted acid catalyst (as for example organic resin). These acid resins lead to a low pH value which is not appropriate for the isomerase enzyme. It is thus necessary to use a liquid membrane linking both compartments, which allows us to perform two sequential reactions of isomerization and dehydration without a cross-limitation between them. However, two main obstacles must be overcome: (i) the thermodynamic equilibrium of the enzymatic isomerization, and (ii) the incompatibility of the pH of the reaction for two catalysts. A methyl isobutyl ketone (MIBC) membrane was chosen in this study, allowing the fructose extraction from the aqueous “donor” phase (Figure 16). D-glucose isomerization was performed at pH = 8 using glucose isomerase. To achieve an efficient transfer through the membrane at room temperature, boronic acid derivatives were used in order to complex the formed fructose at the interface with the “donor” phase. Thanks to Aliquat336^®^, a quaternary amine, an ion pair was formed between the ester with a negatively charged 3,4-dichlorophenylboronic acid (3,4-DCPBA) and fructose [115,116]. Complexed fructose was then transformed to the “receiver” phase containing sulfonic resins, a dehydration catalyst. At pH 3, the fructose complex is hydrolyzed and d-fructose is dehydrated to HMF. An “H” type reactor was implemented, which permitted a better homogenization capacity of the phases. Fructose diffusion was also improved. After 32 h of reaction at a regulated pH (at 8.5 and 3.0) at 70 °C, the extraction yield was 97%. The isomerization yield reached 79% and the HMF yield reached 31% after 32 h of reaction (a glucose conversion of 88%) [115,116].

#### 2.6.3. Hybrid Isomerization

**Figure 17:**
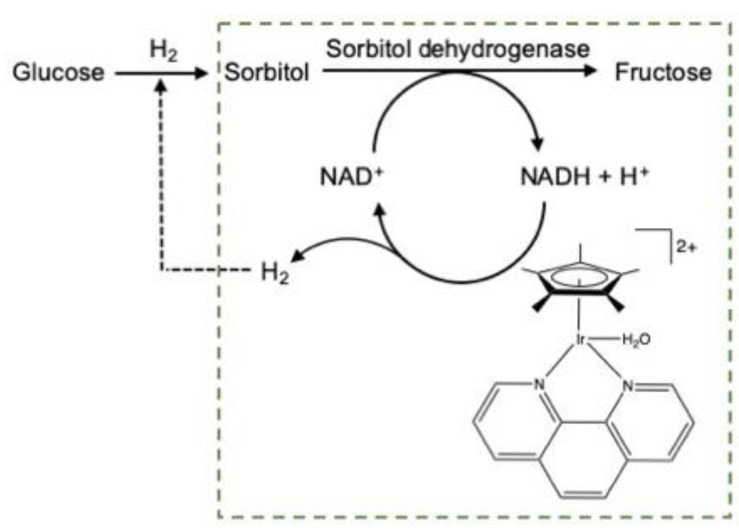
General concept of hybrid catalytic conversion of d-glucose into d-fructose using an iridium complex for NAD+ regeneration.

Another studied variant was based on an innovative enzymatic cascade process. d-glucose is firstly hydrogenated to d-sorbitol, which is then selectively dehydrogenated to d-fructose. It must be noted, however, that the sorbitol dehydrogenase enzyme is NAD+ dependent. The co-substrate must be regenerated throughout the reaction from the NADH formed. This step was carried out using an organometallic Ir-based catalyst (Figure 17).

We demonstrated the compatibility between the enzyme and a chemical catalyst (iridium complex) capable of in situ cofactor regeneration. This proof of concept was patented [114] and the results were published [115]. In addition, the catalyst based on Ir does not seem to constitute a “poison” for the enzyme. We have demonstrated, unambiguously, the continuous catalytic process of the complete system (substrate + cofactor + enzyme + organometallic complex). Up to now, three consecutive catalytic cycles of regeneration of the cofactor with the production of the target molecule were performed. The main challenges of the process concern the optimal operating pH values for both systems that are not compatible yet.

**Figure 18:**
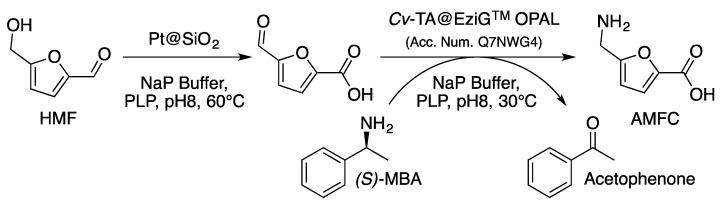
One-pot/two-step hybrid heterogeneous catalytic process for the conversion of HMF into AMFC, combining a Pt/SiO_2_ chemocatalyst and the Cv-TA. Adapted with permission from Ref. [117].

The one-pot approach, which can be described as “true hybrid catalysis”, effectively combines several catalysts within the same reactor, working together in the same conditions. In this way, the various substrates and intermediates are continuously converted as soon as they appear, which can make it possible to shift equilibria, limit inhibitions and thus considerably increase the activities of the catalysts. Recently, we have published an example of the use of hybrid catalysis for the transformation of biomass compounds [117]. This first study of its kind produced a new range of furfurylamines, with a special focus onto 5-aminofuran-2-carboxylic acid (AMFC), directly from HMF (Figure 18). To achieve such a process, for the first time ever reported, a transaminase was coupled with a heterogeneous chemo-catalyst, a platinum nanoparticle immobilized onto silica in this case.

By using a one-pot/two-step process, in which the enzyme was added directly to the reaction medium after the catalyst has completed its reaction and the temperature has dropped to around 30 °C, a 77% yield of FMCA can be achieved. It is noteworthy that the reaction medium was found to be compatible with both catalysts after its optimization and that the only byproduct formed was 2,5-furandicarboxylic acid (FDCA; 23% yield obtained), a highly valuable monomer involved in polyethylene furanoate (PEF) synthesis.

### 2.7. Solving the Contextual Problem of Implementing Solutions

The contributions to this journal concern technical solutions to systemic problems: climate change, pollution, meeting the energy requirements of society, satisfying the market, the conversion of waste into a product to make the economy more circular, or the biodegradation of waste, the development of more refined and tailored materials to meet changing needs in society. The solutions are chemical or biochemical processes. They are innovative, scientifically interesting, if not ground-breaking, and relevant to addressing the systemic problems we face [3].

However, every technical solution, when implemented at the industrial scale, and over enough time, will meet a threshold with regard to causing more harm than good, in the way that we need salt in our bodies, but too much will kill you. In the age of the Anthropocene, we can no longer permit ourselves to implement solutions, at any scale and over any timeframe, that are only determined by demand and the profit of industry [3].

To determine the thresholds, we need to study the context for implementation. The context includes economics, but also society (health, politics, education, workforce, infrastructure, cultural values and security) and the ecology of the location of production, of distribution and of consumption, and then waste disposal. To study all of these elements is complicated. Worse, we cannot afford any longer to let the market work as a massive experiment where we learn through trial and error. As a society, we will have to make compromises and trade-offs. The task of determining what the trade-offs are and how to compare them is necessary, that is, to determine what solutions are worth pursuing and which seem impossible. Additionally, it is with the existing tools that we have: cost–benefit analysis, life-cycle analysis, material and energy flow models, multi-criteria decision aides, environmental impact assessments, cradle to grave analyses. However, these very difficult questions can be answered using an institutional compass [3].

**Figure 19:**
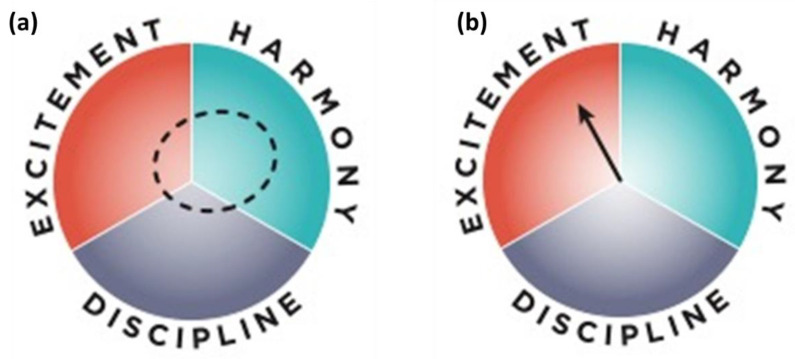
Comparing the wish spot (**a**) with the actual compass reading (**b**).

To make a compass, we gather data about the technological solution process—mainly the input, output and distribution—on economic, social and environmental (entropy, pollution, disease, biodiversity…) levels. We gather data for the proposed region of production, again at the economic, social and environmental levels. We analyze the data categorically along two axes—economics, society or environment—leading towards harmony, and discipline of excitement. The last three are general qualities. Upon reflection, we notice that any object or process will have one that predominates. Prima facie, none is better or worse, although we might have preferences for cultural or other reasons. Our preferred quality-place on the compass is called a wish-spot (Figure 19). We are not thinking in terms of a scale, but in terms simply of the qualities. The wish spot tells of our preference [3].

We then make a further, more refined analysis of each data point that is not involved in the technological solution. We analyze each data point in terms of the angle range within the quality “sector”. This is sensitive work, especially with the social data. There are straightforward methods for the economic and environmental data. We also determine a length of each data point scaled to the radius of the circle to indicate the importance, weight or momentum of the data point. The latter is achieved simply by determining the relative maximum and minimum values. We apply an algorithm that amalgamates all of the data to represent the whole as an arrow on the compass. This represents the general context for the production of the technological solution. We conduct a similar analysis at different scales for the data concerning the biochemical processes, and superimpose them, at different scales on the “general context of production” compass, to see how it fits in to the general context, and more importantly whether it brings the context arrow closer to the wish spot. We can thereby compare different contexts for their suitability at different scales for the proposed solution. We can compare different solutions to each other, for example, making biofuel from human-edible sugar plants or from farm-animal waste. We achieve this holistically and comprehensively, with a sensitivity to cultural values and expectations [3].

It is a shame to misuse the brilliant solutions brought to us by scientific research. It only benefits the discoveries and adds prestige to science when they are implemented responsively by our industrial partners. Thankfully, we can now ensure this better than we could before.

## 3. Discussion

The bioeconomy is a key parameter not only of the French but also of the whole European economy. A turnover value of EUR 2.3 trillion makes the European union (EU) a world leader in the sustainable use of the biobased resources [118]. A sustainable European bioeconomy supports the creation of new bio-based value chains and greener, more cost-effective industrial processes. Research and innovation in the field of new and sustainable bio-based products (biochemicals and biofuels) improve the European capacity to substitute fossil resources. It must be underlined that the sustainable bioeconomy is only a piece of the circular economy. In addition, there is not one single bioeconomy in Europe but several bioeconomies, each with specific local contexts. The chemical industry will be a key player in the European economy. It will allow residues and biowastes to be turned into valuable products which already creates new jobs in the markets of bio-based chemicals, pharmaceuticals and bio-based plastics production, while the employment in the classical fuel sectors decreased. This clearly shows that the use of biomass to produce highly added materials can directly increase employment by 10 times in Europe, and generate 4–9 times more added-value, compared to the current fossil-based economy [118]. As more than 90% of chemical processes are carried out using at least one catalyst (chemical, enzymatic, or heterogeneous), catalysis is in the very heart of the whole chemical industry.

In this context, the research projects developed in the VAALBIO group at UCCS encompass several key directions: biomass fractionation, cellulose [119,120] and hemicellulose valorization [121], green hydrogen production and CO_2_ valorization [122,123,124,125,126,127], and more recently, multicatalytic materials [128] and lignin valorization. As shown above, our research projects concern mainly the synthesis of important chemical synthons using heterogeneous and chemo-enzymatic processes. Some of these synthons are highly valued platform molecules that are building blocks for polymers or pharmaceutical complex molecules. It is worth adding that more than half of the projects are developed in collaboration with industrial partners which clearly makes the VAALBIO group an applied catalysis laboratory. The development of a circular economy also affects our research project’s development. Recently, the VAALBIO group was awarded with a RECABIO “Catalyses and bioeconomy” flagship project from I-SITE ULNE [129]. The RECABIO project is a part of the development of innovative approaches in the field of bioeconomy, and more precisely in the circular economy. It aims at the use of advanced catalytic processes as an alternative to the transformation of renewable resources and wastes to high-added-value molecules, materials and energy carriers. It implements original scientific approaches (in particular, hybrid catalysis, photo- and electro-catalysis as well as their combinations) thanks to (i) the advanced integration of a set of cutting-edge skills from the Lille site, (ii) the use of unique equipment with which our site is equipped (such as the REALCAT platform [130]), and (iii) the hosting of four recognized professors in the field of heterogenous and enzymatic catalysis, human sciences and LCA. The socio-economic and environmental rationalization of the processes thus developed must also be evaluated thanks to the close collaboration between the so-called “hard” sciences and the social sciences (LCA, economics, etc.). In this context, the reception of professors from different fields of science will support these topics, especially with regard to interconnected aspects. This convergence will allow new breakthrough discoveries and will induce an evolution of the concept of bioeconomy from a simple juxtaposition of disciplines (a necessary step that is currently well advanced) to a true integration of scientific and technical disciplines. To achieve this, the present project relies on interdisciplinary research efforts in the hard sciences aiming at lifting the scientific and societal barriers of the bioeconomy, and at questioning the consequences of the ecological mutation and its ethical, socio-political and socio-economic aspects. The ultimate ambition is to create new disciplines as a result of these interfaces, with a view to a global R&D approach, where production systems will be set in their social, political and environmental contexts.

## 4. Conclusions

Catalytic processes are at the heart of chemical transformations. As the VAALBIO team, we study various catalytic processes. We are particularly well known for our work in the field of catalytic valorization of bio-based compounds, but we also have historical expertise in the valorization of light hydrocarbons (and more specifically alkanes) such as methane. These scientific themes, in full expansion, meet the needs of the chemical and energy sectors and will therefore remain at the heart of our team’s concerns for the coming years. The VAALBIO team has skills in chemistry but also in chemical engineering, which allows us to consider the development, not only of innovative catalysts, but also more broadly of catalytic processes as a whole, which constitute a real engine of innovation for the industry of tomorrow. In line with this dynamic, our projects keep a good balance between applied research (mainly supported by industrial partners) and fundamental research (generally supported by institutions). We are also developing an integrated approach to conduct catalytic processes, including the development of innovative contact modes and corresponding catalytic materials as well as processes combining several catalytic functions (hybrid catalysis). Furthermore, very recently, we included the “social” dimension to our project collaborating with experts in human sciences.

## Figures and Tables

**Table 1:** Comparison of furfural oxidation efficiency for Au/HT catalysts with supports of different Mg: Al molar ratios. Reaction: *p* = 6 bar (O_2_); t = 110 °C; 2 h; 600 rpm; FF/Au molar ratio = 200:1; [FF] = 22.7 mM; 25 mg catalyst. Adapted with permission from Ref. [27].

Catalyst	Temperature (°C)	Conversion (%)	FA Selectivity (%)	Carbon Balance (%)
Au/HT 2:1	110	82	86	89
Au/HT 3:1	110	76	88	91
Au/HT 4:1	110	99	100	100
Au/HT 5:1	110	99	100	100

**Table 2:** Effect of the time of 1,6 hexanediol oxidation reaction at 120 °C and 135 °C. Adapted with permission from Ref [36].

T ^a^ (°C)	t ^b^ (h)	X Cha ^c^ (%)	Selectivity ^d^ (%)				
			Chole	Chone	AA	GA	SA
120	4	1.2	84	11	5	0	0
	10	5.2	66	17	13	4	0
135	4	3.7	65	15	18	2	0
	10	12	13	38	33	11	5

^a^ Temperature of reaction (°C). ^b^ Time of reaction (h). ^c^ Conversion of cyclohexane (Cha). ^d^ Selectivities of cyclohexanol (Chol), cyclohexanone (Chone), adipic acid (AA), glutaric acid (GA), and succinic acid (SA).

**Table 3:** Effect of the temperature on FDCA synthesis from K_2_F using Ag/SiO_2_ and CdI_2_ catalysts. Conditions: 17 mg of CdI_2_; 35 mg of K_2_F; 50 mg of Ag/SiO_2_; F_N2_ = 45 mL/min; 20 rpm; T = 200–260 °C; t = 20 h. Adapted with permission from Ref. [72].

Catalyst	Temperature (°C)	Conversion (%)	STY_FDCA_ (µmol kg^−1^ h^−1^)	STY_DFF_ (µmol kg^−1^ h^−1^)
CdI_2_	200	0	-	-
Ag/SiO_2_ + CdI_2_	200	51	264	951
Ag/SiO_2_ + CdI_2_	230	74	145	-
Ag/SiO_2_ + CdI_2_	260	69	188	-
Ag/SiO_2_	200	20	1203	-

**Table 4:** Dynamic kinetic resolution of α-methylbenzylamine catalyzed by hybrid catalyst.

Biocatalyst	Conversion (%)	ee Prod	Productivity ^c^
N435 ^a^	99	>99	0.17
EnzPd_1% ^b^	84	>99	2.21
EnzPd_5% ^b^	76	98	2.00
EnzPd_10% ^b^	78	95	2.17
EnzNoPd ^a^	92	82	2.36

^a^ Reaction conditions: 5% of Pd supported on BaSO_4_ as a racemization agent. A total of 3 mmol of α-methylbenzylamine, 2 eq. of methyl methoxyacetate, biocatalyst (94 mg mmol^−1^ of substrate), molecular sieves (30 mol%, 375 mg), Na_2_CO_3_ (12 mg), 15 eq. of ammonium formate and toluene (3 mL) at 70 °C for 17 h, according to [112]. ^b^ Reaction conditions: 3 mmol of α-methylbenzylamine; 2 eq. of methyl methoxyacetate, biocatalyst (94 mg mmol^−1^ of substrate); molecular sieves (30 mol%, 375 mg); Na_2_CO_3_ (12 mg); 15 eq. of ammonium formate and toluene (3 mL) at 70 °C for 17 h; ^c^ expressed on mg of product h^−1^. mg of immobilized enzyme^−1^.

**Table 5:** DKR of biocatalysts using ammonium formate in continuous-flow condition [112].

Entry	Catalyst (Pd + CALB_imm_)	Reaction Time (h)	Conversion (%)	ee Prod (%)	Selectivity(%)
1	MN@4.5%Pd ^a^ + MN@CALB	1	95	>99	91
2	MN@4.5%Pd + N435 ^b^	4	>99	>99	96
3	Pd/BaSO_4_ + MN@CALB	1	65	>99	84
4	Pd/BaSO_4_ + N435	1	72	97	94
5	MN@4.5%Pd_CALB	9	65	>99	88

^a^ MN@4.5%Pd = Magnetic nanoparticle with 4.5% palladium; MN@ = hybrid catalyst without enzyme; ^b^ N435 = Novozym^®^ 435 (Lipase B de Candida antarcticaimobilized) commercial; Pd/BaSO_4_ = palladium immobilized on barium sulphate (commercial) and MN@4.5%Pd_CALB = optimized hybrid catalyst.

## Data Availability

Not applicable.
